# Supplementary material for: Research on the NI-MLA Method for Enhancing the Spot Position Detection Accuracy of Quadrant Detectors Under Atmospheric Turbulence
Source: Sensors (Basel). 2024 Oct 17;24(20):6684. doi: 10.3390/s24206684 (PMC11510952; doi:10.3390/s24206684)
Supplement: Supplementary file 1 [file sensors-24-06684-s001.zip › sensors-3233990-supplementary.pdf]

ANGULAR MEASUREMENT CHARACTERISTIC OF THE IMAGING MICROLENS ARRAY (I-MLA)

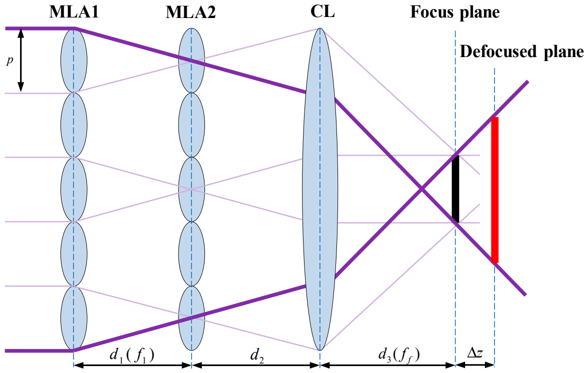

Figure S1. Schematic diagram of NI-MLA system.

The y_o and θ_o represent the beam's height of image plane and outgoing angle respectively on the image plane of the system shown in Fig.A1 can be expressed as:

$$\begin{bmatrix} y_o \\ \theta_o \end{bmatrix} = \begin{bmatrix} -(f\cdot\theta_i + np)(\frac{d_3}{f_f}-1) - \frac{y_i}{f}[d_3 - d_2(\frac{d_3}{f_f}-1)] \\ \frac{y_i}{f}(\frac{d_2}{f_f}-1) - \frac{f\cdot\theta_1 + np}{f_f} \end{bmatrix} \tag{S1}$$

The system focal length F is

$$F = \lim_{\Delta\theta_1 \to 0} \frac{\Delta y_o}{\Delta\theta_i} = -f(\frac{d_3}{f_f}-1) \tag{S2}$$

When $d_3 = f_f$, the focal length F of the NI-MLA system is $F = 0$, that is, when the image plane is located in the focal plane of the condenser lens, even if the incident angle changes within a certain range, the spot position on the image plane remains unchanged. So, the height of the emitted light is independent of the incident angle. From this, it can be concluded that the I-MLA system does not possess angular measurement characteristics at $d_3 = f_f$.When $d_3 = \Delta z + f_f$, that is, the image plane is located at the defocus of CL, the focal length of I-MLA system is $F = -f\,\Delta z / f_f$.It can be observed that, although y_o is proportional to θ_o within the paraxial range, the focal length of the I-MLA system is quite small (for example, when $f = 5$ mm, $f_f = 25$mm and $\Delta z = 1$mm, the focal length of system is $F = 0.2$ mm). However, in the fields of semi-active laser guidance and laser communication, the focal length of QD optical systems typically ranges from tens to hundreds of millimeters.

Therefore, because there is no focal length on the focal plane of CL for the I-MLA system and its focal length is too small when defocused, this system is not suitable for angular measurement applications in these fields.
